# Lime-Based Sorbents for High-Temperature CO_2_ Capture—A Review of Sorbent Modification Methods

**DOI:** 10.3390/ijerph7083129

**Published:** 2010-08-06

**Authors:** Vasilije Manovic, Edward J. Anthony

**Affiliations:** CanmetENERGY, Natural Resources Canada, 1 Haanel Drive, Ottawa, Ontario, K1A 1M1, Canada; E-Mail: banthony@nrcan.gc.ca (E.J.A.)

**Keywords:** CO_2_ capture, Ca looping cycles, thermal pre-treatment, hydration, pelletization

## Abstract

This paper presents a review of the research on CO_2_ capture by lime-based looping cycles undertaken at CanmetENERGY’s (Ottawa, Canada) research laboratories. This is a new and very promising technology that may help in mitigation of global warming and climate change caused primarily by the use of fossil fuels. The intensity of the anticipated changes urgently requires solutions such as more cost-effective technologies for CO_2_ capture. This new technology is based on the use of lime-based sorbents in a dual fluidized bed combustion (FBC) reactor which contains a carbonator—a unit for CO_2_ capture, and a calciner—a unit for CaO regeneration. However, even though natural materials are cheap and abundant and very good candidates as solid CO_2_ carriers, their performance in a practical system still shows significant limitations. These limitations include rapid loss of activity during the capture cycles, which is a result of sintering, attrition, and consequent elutriation from FBC reactors. Therefore, research on sorbent performance is critical and this paper reviews some of the promising ways to overcome these shortcomings. It is shown that reactivation by steam/water, thermal pre-treatment, and doping simultaneously with sorbent reforming and pelletization are promising potential solutions to reduce the loss of activity of these sorbents over multiple cycles of use.

## 1. Introduction

It is widely accepted that climate change is being exacerbated by increasing atmospheric concentrations of greenhouse gases, and this is a key problem that requires urgent solutions. Fossil fuel combustion electrical generation plants represent a major source of anthropogenic CO_2_. About a third of global CO_2_ emissions come from the burning of fossil fuels in electricity production. Reduction of such emissions may significantly decrease total emissions of greenhouse gases. One possible approach is the capture of CO_2_ from flue gas followed by its sequestration in geological formations [1–3]. The purpose of CO_2_ capture is to produce a concentrated stream of CO_2_, preferably at high pressure, so that it can readily be transported to a storage site. Although, in principle, the entire gas stream containing low concentrations of CO_2_ could be transported and injected underground, the energy requirements and other associated costs generally make this approach impractical.

The capture step for CO_2_ from large point sources is a critical one with respect to the technical feasibility and cost of the overall carbon sequestration scenario. CO_2_ separation is the first and most technically challenging and energy intensive step; therefore, much research has been targeted at improving current technologies or developing new approaches to CO_2_ separation. Important new classes of technologies for CO_2_ separation are based on solid looping cycles [4]. Two types of solid looping cycles for CO_2_ separation are chemical-looping combustion (O_2_ cycles) and CaO-based CO_2_ looping cycles. The common characteristic is the use of solids that circulate between two different chemical environments with fluidized bed combustion (FBC) systems as an optimal technology.

**Figure 1.**
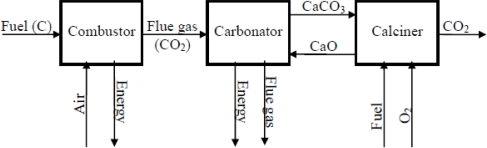
Schematic representation of CaO-based CO_2_ looping cycles.

A looping cycle process, which employs a solid CaO-based carrier, is schematically presented in Figure 1. It may inexpensively and effectively remove CO_2_ from combustion gases, allowing it to be regenerated as a pure CO_2_ stream suitable for sequestration [4,5]. The use of solids also means that, in many cases, FBC systems represent optimal technology for such processes [6–8] since they permit large amounts of solids to be transferred easily from one chemical environment to another [9]. The deployment of such technologies has the added advantage that both large (>350 MWe) atmospheric and pressurized systems also exist [10,11], and so the technical challenges of developing such systems for a number of possible schemes are significantly reduced. Preliminary economic analyses [12–14] suggest that such processes are economically attractive, and an important advantage of using CaO is that limestone is abundant and a relatively inexpensive material.

CO_2_ capture by CaO-based sorbents is based on the reversible chemical reaction (carbonation/calcination): CaO_(s)_ + CO_2(g)_ = CaCO_3(s)_. CO_2_ separation from flue gas is possible in a multi-cycle process in a dual reactor. This involves reaction of CaO with CO_2_ from flue gas in a carbonator, and regeneration of sorbent in a calciner [8]. In the ideal case, carbonation/calcination cycles can be carried out indefinitely with the only limitations due to the kinetics of the reactions and thermodynamics of the equilibrium system. The use of the carbonation reaction (exothermic) is limited by the maximum temperature that allows CO_2_ capture at the desired concentration in cleaned flue gas and the minimum temperature that allows a practical reaction rate. The calcination reaction is limited by the minimum temperature necessary to obtain sufficient CO_2_ concentration at the calciner outlet.

Despite the simple chemistry of carbonation/calcination looping cycles, undesirable side reactions such as sulphation and processes such as sintering and attrition take place in practice. SO_2_ from flue gas under CO_2_ looping cycle conditions irreversibly reacts with CaO, forming CaSO_4_. A portion of the CaO sorbent is, therefore, lost as CaSO_4_, and the CaO reaction surface is covered by this product, preventing contact of CaO and CO_2_ [15,16]. Attrition of sorbent is a significant problem for FBC systems, leading to sorbent elutriation from the reactor [17]. The major and most investigated challenge for CO_2_ looping cycles is the decrease of reversibility for the carbonation reaction due to sorbent sintering [18,19].

**Figure 2.**
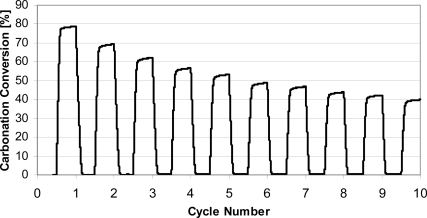
Loss of sorbent (Cadomin limestone, 250–425 μm) activity during carbonation/calcination cycles in TGA; 700 °C isothermally: 60 min carbonation in 15% CO_2_ (N_2_ balance), 60 min calcination in N_2_.

A typical TGA multi-cycle run with natural limestone is presented in Figure 2. It can be seen that after only 10 cycles, conversion dropped to 40%, half the conversion in the first cycle. The loss of activity continues, and it has been shown in long series of cycles (>1,000 cycles) that conversions become constant, at the level of 7–8% [20]. It should also be mentioned that most research has been performed under ideal experimental conditions, with calcination stages in N_2_ at lower temperatures, while the loss of activity occurs much faster under realistic conditions expected in real FBC systems [21,22]. It is typically supposed that during CO_2_ cycles, the sorbent morphology changes, and the sorbent loses surface area and small pores, which are the main contributors to the rapid carbonation necessary for practical systems.

The improvement of sorbent activity for extended use is imperative because sorbent replacement costs strongly influence the overall cost of CO_2_ capture [14]. Reactivation by hydration currently appears to be a promising method for recovery of the sorbent activity [15,23,24]. Another approach is thermal pretreatment of the sorbent at high temperatures [25–27]. Here, thermal pretreatment typically causes lower conversions during the early cycles, but conversions in later cycles are higher than those for the original, untreated sorbents. This phenomenon of increasing conversion with an increasing number of reaction cycles has been called self-reactivation [26].

Most doping agents enhance sintering, which is also enhanced by the presence of impurities in the sorbent [22,28]. However, it has been shown that some compounds in the CaO structure prevent sintering. The most investigated are synthetic sorbents with Al_2_O_3_ [29–31], but recently it has been shown that sorbents containing KMnO_4_ [32], and TiO_2_ [33] are also more resistant to sintering. Other attempts include modification by acetic acid [34], and impregnation of CaO on mesoporous supports [35]. Commercial pure lime nano-sized sorbents [36] or flame-made doped nano-particles [37] were also investigated. Nano-sized sorbents were prepared from different precursors [38,39] and procedures [27,40,41]. These modified/synthetic sorbents have superior CO_2_ carrying capacity, but their practical use is limited due to their high price and difficulties for their use in FBC systems.

Therefore, in our laboratory at CanmetENERGY we have put emphasis on procedures and materials which could enable commercially competitive CO_2_ capture by reactivated, modified, or synthetic/pelletized sorbents. We have intensely investigated spent sorbent reactivation by steam, sorbent pretreatment at elevated temperatures, and doping/pelletization with commercial calcium aluminate cements. The results of these investigations are presented in this paper with an emphasis on calcium aluminate pellets and their suitability for reactivation and reforming.

## 2. Methodology

A number of limestones were tested, differing in chemical composition, geological origin and geographical location. Kelly Rock, Cadomin (formerly called Luscar), Graymont (GR) and Havelock (HV) are Canadian limestones; La Blanca is Spanish limestone, and Katowice is Polish (Upper Silesia) limestone. Their composition can be found in our published papers [15,22,23,31]. Four commercial cements were used for pelletization. CA-14 and CA-25 were produced by Almatis Inc., and have Al_2_O_3_ contents of 70 and 80%, respectively. Secar 51 and Secar 80 are produced by Kerneos Inc., and were chosen because of their wider difference in Al_2_O_3_ content, 50 and 80%, respectively. They are produced in large quantities and are relatively inexpensive, and are used when refractory properties—resistance to corrosion and chemicals—and rapid setting are required [42]. This also recommends them as potential binders for CaO-based pellets for CO_2_ capture. The limestone/cement ratio was 9:1, and more details on the pellet preparation procedure can be seen elsewhere [31].

Three main aspects of sorbent performance enhancement were tested in our studies:

- In the steam reactivation tests, limestone samples were subjected to calcination/carbonation cycles and reactivated in a pressurized steam reactor [15].
- Thermal pretreatment of samples has been done in a tube furnace [43] or directly in a thermogravimetric analysis apparatus (TGA) [24] before cycles.
- Original or spent sorbents were pelletized using calcium aluminate cements as binders [31].

A Perkin Elmer TGA-7, or a Mettler Toledo TGA/SDTA851^e^/LF/1100 °C TGA was usually used for the experiments. The sample was in a ceramic or platinum pan (5 mm diameter). The gas flow rate, controlled by a flowmeter, was 0.04 dm^3^/min. Calcination/carbonation cycles were carried out under different conditions. The calcination stages were done under milder conditions in an N_2_ atmosphere [15,23,25,26,31,43], or at higher temperature in pure CO_2_, simulating real sorbent regeneration conditions [21,44,45].

The samples were examined by scanning electron microscope (SEM). A Hitachi S3400 microscope with 20 kV of accelerating voltage was used. The samples were usually coated with a 3 nm thick layer of gold-palladium.

## 3. Results and Discussion

### 3.1. Steam Reactivation

Our research on spent sorbent reactivation was based on experience with reactivation of sorbent utilized for SO_2_ retention [46–48]. Carbonation, like sulphation, is a gas-solid reaction with solid product formation at the surface of the reactant; therefore, similar limits and methods for reactivation were expected. An important difference is reversibility of carbonation, *i.e*., the product layer may be easily removed to expose sintered sorbent surface area to hydration. Taking into account the above analysis, sorbent hydration appears to be the most promising method for reactivation. It is based on a simple chemical reaction: CaO + H_2_O → Ca(OH)_2_. Considering the hydration technique, hydration by steam was chosen because there is no excess water to be removed, sample drying is not required and there is no loss of sorbent with liquid reactant [15].

**Figure 3.**
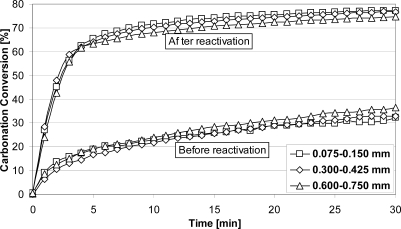
Steam reactivation effect on sorbent activity during carbonation in the TGA [15]. Kelly Rock limestone; 20 cycles (20% CO_2_, N_2_ balance, 650 °C, 30 min/100% N_2_, 850 °C, 30 min); reactivation by steam (saturated steam, 200 °C, 30 min); and carbonation in TGA (15% CO_2_, N_2_ balance, 700 °C).

Typical results for sorbent reactivation tests are presented in Figure 3. It may be seen that spent sorbent had a carbonation degree ∼35%, regardless of particle size. After reactivation, the sorbent had higher activity and final value for the carbonation conversions in the first cycle above ∼75%, regardless of the particle size, which was significantly higher than related values for spent sorbent.

**Figure 4.**
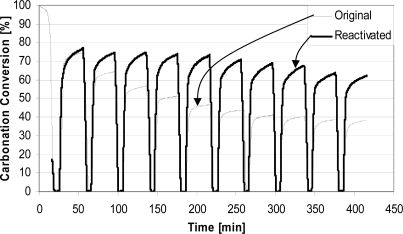
Steam reactivation effect on sorbent activity during CO_2_ cycles in the TGA. Kelly Rock, 0.300–0.425 mm; calcination (100% N_2_, 850 °C); carbonation (15% CO_2_, N_2_ balance, 650 °C).

The behaviour of reactivated spent sorbent in CO_2_ capture cycles is shown in Figure 4. It can be seen that carbonation in the initial cycles was higher than for the natural sorbent and reactivated sorbent displayed significantly better conversions at the end of multi-cycle tests. The final result is an average carbonation of ∼70% during 10 cycles with reactivated sorbent. This analysis shows that steam reactivation actually improves sorbent characteristics, and may enable use of the sorbent for prolonged times, or at least until attrition phenomena dominate. This can aid in further development of the process of CO_2_ separation by CaO-based sorbents.

### 3.2. Thermal Pretreatment

**Figure 5.**
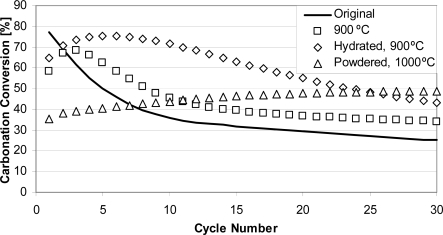
The effect of sorbent preheating for 24 h on its CO_2_ carrying capacity in TGA [26]. Kelly Rock, 0.300–0.425 mm; calcination (100% N_2_, 10 min); carbonation (50% CO_2_, N_2_ balance, 30 min); isothermally at 800 °C.

The main idea behind thermal pretreatment is to stabilize sorbent morphology, which aids in maintaining sorbent CO_2_ carrying activity along cycles. CO_2_ looping cycles were performed with samples pretreated at different temperatures (800–1,300 °C) for different durations (6–48 h). It can be seen in Figure 5 that the sample treated at 900 °C had ∼20% lower conversion in the first cycle than that of the original sample. However, conversion for the pretreated sample increased and in the third cycle was ∼8% higher than that of the original sample. In subsequent cycles, conversion was typically at least 10% greater than that of the original untreated sorbent. Also, experiments performed with pretreated hydrated samples show significant increase of conversions for the initial 6–7 cycles. This effect we have called self-reactivation. Perhaps the most interesting result is seen for powdered samples (<50 μm). It can be seen that self-reactivation occurred for the entire 30 cycles, and the highest conversion was obtained for the last cycle (49%). This finding was supported by experiments with four Canadian limestones [26]. However, it should be mentioned that there are sorbents that do not show enhanced performance after pretreatment at high temperatures, and La Blanca (Spanish) limestone is one such example [49].

### 3.3. Calcium Aluminate-Based Pellets

Calcium aluminate-based pellets are new and one of the most efficient and inexpensive solid sorbents for CO_2_ capture [31]. They can help in mitigation of sorbent sintering, attrition, and consequent elutriation. All of these shortcomings are considered to be mitigated by means of reactivation/pelletization of fresh/spent/elutriated sorbent before or during its utilization. It has been shown that the use of appropriate binders is necessary and calcium aluminate cement is the most efficient binder [50]. It is also a source of alumina compounds desirable in the CaO structure, which enhance micro- and nano-porosity of the sorbent. However, like other CaO-based sorbents, aluminate-based pellets lose their activity, which is especially pronounced at higher temperatures [44] necessary during sorbent regeneration in order to produce concentrated CO_2_ streams.

**Figure 6.**
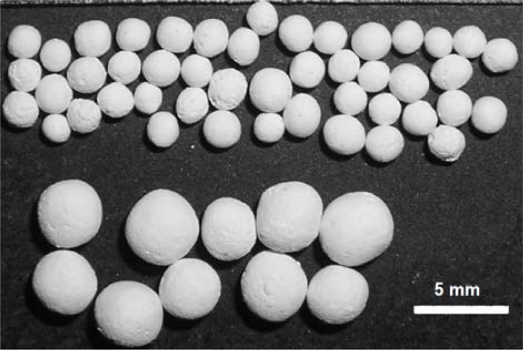
Photograph of calcium aluminate-based pellets.

**Figure 7.**
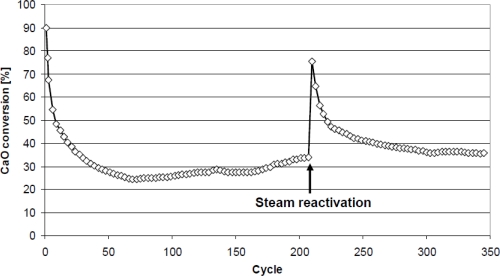
Carbonation conversion of reformed aluminate-based pellets (1 mm) enhanced by steam reactivation (5 min at 100 °C in saturated steam). Pellets were prepared with 10% CA-14 cement and 90% Cadomin limestone [31]. Calcination at 950 °C in 100% CO_2_; carbonation at 700 °C in 20% CO_2_ (N_2_ balance) for 30 min.

The reactivation of aluminate-based pellets by steam or water to recover their activity during capture cycles has also been investigated [45]. Moreover, these pellets can be reshaped after reactivation by water, which is another advantage. A photograph of pellets is presented in Figure 6. A test involving 300 cycles in a tube furnace, followed by reactivation and reshaping of the pellets, was continued for a further 350 cycles in the TGA. During this series of 350 cycles, a steam reactivation step was applied after 210 cycles. The CO_2_ capture activity of pellets was determined in a TGA apparatus, and Figure 7 presents TGA results from this 350-cycle test. These results illustrate the superior performance of aluminate-based pellets, with the added important property that they can be reactivated/reformed, resulting in high average conversions, >35%, in series of hundreds of CO_2_ capture cycles.

**Figure 8.**
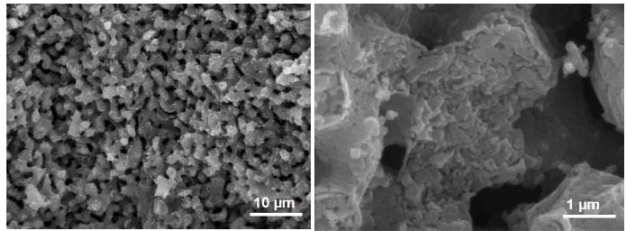
Morphology of aluminate-based pellets (SEM).

The morphology of pellets was observed by a SEM and obtained images at magnifications of 2,500× and 20,000× are presented in Figure 8. The images were taken from the interior of broken pellet spheres after 30 CO_2_ cycles. It can be seen that two types of pores are present: large macropores on the 1 μm scale and mesopores on the 10–100 nm scale. These nano-sized pores, which did not disappear during cycles, are responsible for carbonation conversion because they are the main contributor to the sorbent surface area and to the micro- and meso- porosity necessary for storage of more voluminous product, CaCO_3_.

The encouraging property of the pellets prepared here is the high particle strength, noted during handling, which is suitable for FBC systems. The use of inexpensive natural materials such as limestone (∼$10/t) or spent lime-based sorbent provides very low costs for these pellets. The calcium alumina cements are also relatively inexpensive when used at the industrial scale ($1,200–1,300/t). Moreover, these pellets are suitable for reactivation by water and reuse, which additionally highlight their superior performance.

## 4. Conclusions

Solid looping cycles are a rapidly developing technology for CO_2_ capture. When considering thermodynamics and sorbent costs, CaO obtained from limestone is the best candidate for use as a solid carrier of CO_2_ from dilute gases to concentrated streams. The key technology costs are strongly connected with the behaviour of sorbent in cycles. The main hurdles for the technology are overcoming the loss and deactivation of sorbent through sintering and attrition. The intensive research at CanmetENERGY to improve sorbent performance was reviewed in this paper. To date, the most promising methods were reactivation by steam, thermal pretreatment, and pelletization with aluminate-based cements. Based on the research reviewed here, the combination of these methods appears to provide enhanced sorbent performance.
